# Generation of the Bovine Viral Diarrhea Virus E0 Protein in Transgenic *Astragalus* and Its Immunogenicity in Sika Deer

**DOI:** 10.1155/2014/372503

**Published:** 2014-05-22

**Authors:** Yugang Gao, Xueliang Zhao, Pu Zang, Qun Liu, Gongqing Wei, Lianxue Zhang

**Affiliations:** College of Traditional Material Medicine, Jilin Agricultural University, Changchun 130118, China

## Abstract

The bovine viral diarrhea virus (BVDV), a single-stranded RNA virus, can cause fatal diarrhea syndrome, respiratory problems, and reproductive disorders in herds. Over the past few years, it has become clear that the BVDV infection rates are increasing and it is likely that an effective vaccine for BVDV will be needed. In this study, transgenic *Astragalus* was used as an alternative productive platform for the expression of glycoprotein E0. The immunogenicity of glycoprotein E0 expressed in transgenic *Astragalus* was detected in deer. The presence of pBI121-E0 was confirmed by polymerase chain reaction (PCR), transcription was verified by reverse transcription- (RT-) PCR, and recombinant protein expression was confirmed by ELISA and Western blot analyses. Deer that were immunized subcutaneously with the transgenic plant vaccine developed specific humoral and cell-mediated immune responses against BVDV. This study provides a new method for a protein with weak immunogenicity to be used as part of a transgenic plant vaccine.

## 1. Introduction

Bovine viral diarrhea (BVD) is a widespread disease that affects cattle [1]. The causative agent is the bovine viral diarrhea virus (BVDV), a* Pestivirus* of the family Flaviviridae. BVDV infection presents a wide spectrum of diseases, ranging from mild acute infection to fatal mucosal disease [2]. The virus is known to damage the immune system of the infected animals, which can make the animals more susceptible to other diseases and causes significant loss to the livestock industry [3–5]. The virus can infect and be transmitted between a variety of animals, such as cattle, sheep, and whitetail deer [6–9]. In our previous study, a new single strain of BVDV named CCSYD was isolated and verified from sika deer [10].

Different strategies are available to control the spread of BVDV in a herd, such as vaccination and test-and-cull schemes. Among these strategies, test-and-cull schemes have been very successfully applied in areas with low cattle densities and low BVDV prevalence, such as Scandinavia [11, 12]. But in the areas with high cattle densities and high BVDV prevalence, the program will bring huge economic loss. Vaccine research is considered to be a promising alternative to prevent BVDV spread. Inactivated vaccines generally possess inadequate efficacy and do not induce sufficient protective immunity. Although modified live vaccines provide certain protection against homologous strains, the intrinsic risk of virulence reversion remains a concern [13, 14]. Due to the poor immunogenicity of inactivated vaccines and the safety concerns surrounding the use of modified live vaccine, an effective subunit vaccine for BVDV has become the subject of increasing research interest [15].

BVDV is a single-stranded RNA virus and has approximately 12.5 kb RNA genome [16] and the genomes of BVDV are translated and processed into eleven mature proteins. After infection or vaccination, cattle elicit antibodies to the three envelope proteins E0, E1, and E2 and against the nonstructural protein NS_3_ [17]. Glycoprotein E2 is the major target of the protective immune response triggered against BVDV infection and is widely used for subunit vaccines [18, 19]. However, the highly variable sequence of E2 protein often leads to immune failure [20, 21].

E0 is a conserved protein and shows less antigenic diversity than E2 [22]. Nevertheless, the BVDV E0 expressed in prokaryotes system produced neutralizing antibodies but at low titers that could not efficiently neutralize virus, which was attributed to a misfolding of E0 [17]. In view that eukaryotic expression could maintain the correct folding and glycosylation of proteins, eukaryotic expression has become the research focus in the study of subunit vaccine. Last year, Gao et al. [23] successfully constructed a prokaryotic expression vector PVAX1-E0 and identified its antigen activity in rabbits. The result shows that the recombined PVAX1-E0 could produce specific humoral and cellular immune response in rabbits. Transgenic plants are new eukaryotic expression-delivery systems that have become attractive bioreactors in the production of high-value medical peptides and proteins [24]. Up to now, several types of plant species have been used as antigen-delivery systems for subunit vaccines [25, 26]. For example, the truncated glycoprotein BVDV E2 has been expressed in* N. tabacum* leaves and subsequently showed high reactivity in virus neutralization test [27].

Another strategy to improve the immune activity of vaccine is the use of adjuvant [28]. Vaccine adjuvant can stimulate the immune system to an increased specific antibody response.* Radix Astragali *(*Astragalus*) is a plant used as a traditional herb medicine in China, and it is known for its antiviral activity and immunity-boosting properties [29–31].* Astragalus* polysaccharides can improve the function of macrophages, enhance macrophage phagocytosis, and increase the activity of natural killer (NK) cells [32].* Astragalus* as host plants can play a role in the immune adjuvant to enhance the immune level.

In this study, plant expression vector pBI121-E0 was constructed and transferred into* Astragalus*. The immunogenicity and protective efficacy of the recombinant proteins were demonstrated in deer. The aim of this study was to develop a transgenic* Astragalus* vaccine for bovine viral diarrhea.

## 2. Materials and Methods

### 2.1. Reagents, Bacteria, and Plasmids

The plasmid pMD18-T-E0 was developed in our previous work [23]. The* E. coli* strain DH5*α* was purchased from Invitrogen (Shanghai, China). Plasmid pBI121 (Novagen, Darmstadt, Germany) was used for recombinant protein expression. Restriction enzymes, Taq DNA polymerase, TriPure Kit, and T4 ligase were purchased from TaKaRa Biotechnology Co., Dalian, China.

### 2.2. Plasmid Construction

The BVDV E0 open reading frame was amplified from plasmid pMD18-T-E0 which contains the complete gene with the forward primer (5′-CCG GAT CCA CCA TGG AAA ACA TAA CAC AGT GG-3′,* BamHI* site underlined) and the reverse primer (5′-GCG AGC TCT TAA GCG TAT GCT CCA AAC CAC GT-3′,* SacI* site underlined). The PCR product was digested with* BamHI* and* SacI* and inserted into plant expression vector pBI121 digested with the same enzymes to create the recombinant plasmid pBI121-E0.

### 2.3. Plant Transformation and Genetic Analysis

The* Astragalus membranaceus *(Fisch.) Bungevar.* mongholicus *(Bunge) P. K. Hsiao cultivated in our laboratory was used as the host plant. The transformation experiment was carried out in the middle of July. After 20 h artificial pollination [33], 150 *μ*L plasmid pBI121-E0 (1-2 ng/*μ*L) was applied to the stigmas evenly using a micropipette. At maturity stage, seeds were harvested and stored at room temperature. The transgenic seedlings were identified by PCR and RT-PCR analysis with the forward primer 5′-CCG GAT CCA CCA TGG AAA ACA TAA CAC AGT GG-3′ and the reverse primer 5′-GCG AGC TCT TAA GCG TAT GCT CCA AAC CAC GT-3′.

### 2.4. Protein Extraction

Protein isolation was conducted using 0.5 g fresh leaves of regeneration seedlings that were quickly ground in liquid nitrogen. The resulting powder was resuspended in 1 mL extraction buffer (0.24 g/L KH_2_PO_4_, 1.44 g/L Na_2_HPO_4_, 0.2 g/L KCl, 8 g/L NaCl, 10 *μ*g/mL leupeptin, 5 mM PMSF, 50 mM EDTA, and pH 7.0–7.2) as previously described [27]. The mixture was centrifuged at 12,000 g for 20 min at 4°C, and the protein concentration was measured by the Bradford protein assay [34] in 1 mL of the supernatant using bovine serum albumin (BSA) as a standard.

### 2.5. Enzyme-Linked Immunosorbent Assay (ELISA)

To illustrate the expression of glycoprotein E0, the ELISA was carried out. ELISA assay plates were coated at 4°C overnight with 90 *μ*L protein samples (containing 30 *μ*g total protein extracted from transgenic* Astragalus*) diluted in 10 *μ*L coating buffer (pH = 8.0). After removing the liquid, the plates were washed three times with phosphate-buffered saline (PBS) with Tween 20 (PBST) and blocked with 10% horse serum at 37°C for 1 h. Then, BVDV-positive bovine serum (1 : 20 dilution) was added to the plates and incubated for 1 h at 37°C. Then, the plates were incubated for 1 h at 37°C with horseradish peroxidase (HRP) conjugated rabbit anti-bovine IgG (1 : 5,000 dilution) as the secondary antibody. Substrate was added for colour reaction at 37°C for 15 min and reaction was terminated by adding 2 M H_2_SO_4_ (50 *μ*L/well). The absorbance was examined using an ELISA reader at 490 nm.

### 2.6. Western Blot Analysis

To further demonstrate the immune activity of the glycoprotein E0 expressed in transgenic lines, the recombinant proteins were detected by Western blot analysis. The total soluble proteins (60 *μ*g) extracted from fresh leaves of transgenic* Astragalus* were subjected to electrophoresis on a 12% sodium dodecyl sulfate polyacrylamide gel (SDS-PAGE) and transferred to a nitrocellulose membrane (GE Healthcare, USA). Western blot was carried out using BVDV-positive bovine serum (1 : 100 dilution) and HRP-conjugated rabbit anti-bovine IgG (1 : 5000 dilution, Southern Biotechnology, USA) as the primary and secondary antibodies, respectively. The total proteins were extracted from wild-type* Astragalus* as negative control and glycoprotein E0 was expressed in the baby hamster kidney- (BHK-) 21 cells [23] as a positive control.

### 2.7. Ethics Statement

All the deer were obtained from DongDa Deer Industry Co., Ltd. The deer care and maintenance at the sika deer farm of Jilin Agricultural University and permission to undertake this work were granted by the Management Bureau of Animal Husbandry in Jilin Province (Shaoxian Liu, Director). The research was also approved by the animal ethics committees of Jilin Agricultural University and the National Deer Industry Association of China Animal Agriculture Association (CAAA). We anesthetized all deer prior to sampling. The blood samplings were performed by veterinarians, biologists, or technicians with previous training and experience in these procedures. We collected up to 2 mL of blood via the jugular vein. All surgery was performed under sodium pentobarbital and all efforts were made to minimize suffering. Finally, all the deer were not sacrificed.

### 2.8. Immunization Schedule

Thirty healthy one-month-old young male sika deer were randomly divided into five groups (six deer per group). The vaccines were prepared as previously described [27]. Briefly, montanide ISA 70 SEPPIC and Al (OH)_3_ hydrogel were used as the adjuvant in oil vaccine and in aqueous vaccine. And the ratios of antigen to adjuvant were 40 : 60 and 90 : 10, respectively. The deer in group one were immunized subcutaneously with 1 mL formulated oil vaccine containing 100 *μ*g total protein extracted from transgenic* Astragalus*; group two was immunized s.c. with 1 mL formulated aqueous vaccine containing the same dose of the protein extracted from transgenic* Astragalus*. Group three and group four acted as negative groups; deer in the two groups were immunized s.c. with 1 mL oil vaccine and aqueous vaccine containing 100 *μ*g total protein extracted from wild-type* Astragalus*, respectively. Group five was injected s.c. with 100 TCID_50_ of inactivated BVDV vaccine (purchased from Chinese Veterinary Drug Control Room). The second immunization was carried out in all groups on day 14. Blood samples were collected at the time of immunization (day 0) and on the days 7, 14, 21, 28, 35, and 42 after the first immunization. The blood sera were used for lymphocyte blastogenesis assay and ELISA assay.

### 2.9. Determination of Specific Antibodies in Immunized Deer

The blood taken from sika deer was diluted (1 : 40) with coating buffer (pH = 8.0) and added into microtiter plates (100 *μ*L/well). The microtiter plates were coated for 2 h at 37°C. After removing the liquid, the plates were washed three times with PBST and blocked with 10% horse serum for 2 h at 37°C. Then, 100 *μ*L soluble whole virus antigen (containing 100 *μ*g viral proteins) extracted from the C_24_V BVDV (purchased from the China Institute of Veterinary Drug Control) was added to the plates and incubated for 2 h at 37°C. The plates were then washed three times with PBST and incubated for 1 h at 37°C with 100 *μ*L BVDV-positive bovine serum (1 : 200 dilution). HRP-conjugated rabbit anti-bovine IgG (1 : 5000 dilution) was used as the secondary antibody. Substrate was added to facilitate the color reaction at 37°C for 15 min and the reaction was terminated by the addition of 2 M H_2_SO_4_ (50 *μ*L/well). The optical density (OD) was assessed at 490 nm.

### 2.10. Lymphocyte Blastogenesis Assay

Peripheral blood mononuclear cells were isolated from anticoagulated jugular blood as previously reported (see Figure 4) [35]. More than 95% of the cells were lymphocytes. The cell viability was checked with a trypan blue dye exclusion assay and the cells were resuspended in Roswell Park Memorial Institute medium (RPMI) 1640, supplemented with 10% fetal bovine serum, 100 IU/mL penicillin (Sigma), and 100 mg/mL streptomycin (Sigma). The cells were placed into 96-well round-bottom plates at a concentration of 4 × 10^6^ cells/well (100 *μ*L) and incubated with or without BVDV (10^4^ TCID50/well) in hexaplicate at 37°C under 5% CO_2_. Phytohaemagglutinin(PHA) (Sigma) at 5 mg/mL was used as a positive control. After 40 to 48 h incubation at 37°C under 5% CO_2_, 3-(4,5-dimethylthiazol-2-yl)-2, 5-diphenyltetrazolium bromide (MTT) was added to each well and the cells were cultured for 4 h. After the addition of 100 *μ*L dimethyl sulfoxide (DMSO) to each well to stop the colour development, plates were tested at 570 nm.

### 2.11. Statistical Analysis

Multiple group comparisons were performed using one way analysis of variance (ANOVA) followed by Tukey's test in order to detect intergroup differences. GraphPad Prism software (Version 5.0; GraphPad Software, Inc., San Diego, CA) was used to perform the statistical analysis. A value of *P* < 0.05 was considered statistically significant.

## 3. Results

### 3.1. Genetic Identification of the Transgenic Astragalus

**Figure 1:**
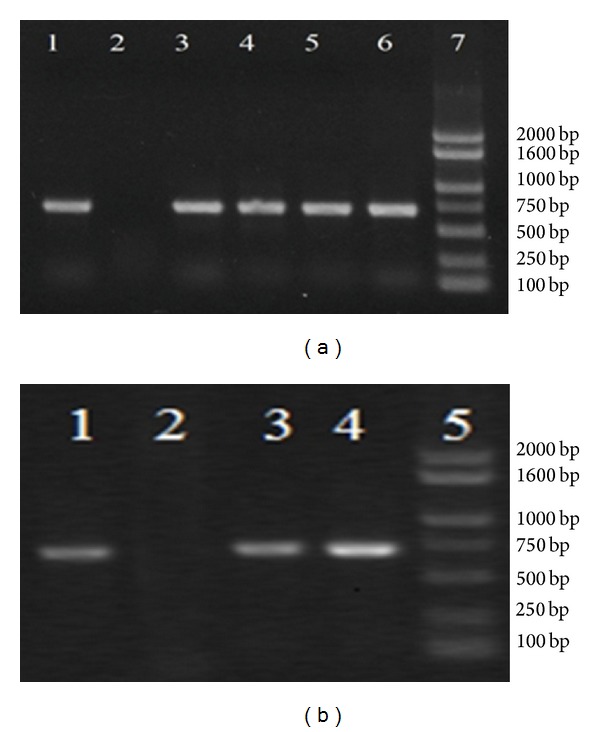
Genetic identification of the transgenic* Astragalus.* The genomic DNA and total RNA were extracted from the leaves of transgenic* Astragalus *for PCR (a) and RT-PCR (b) identification, respectively. (a) PCR analysis of the transgenic* Astragalus*. Total genomic DNAs were extracted from the leaves of different transgenic* Astragalus* spp. (lanes 3–6) and untransformed wild-type* Astragalus* (lane 2 as negative control). Lane 1 is indicated as the positive control by using pBI121-E0 plasmid DNA as PCR template. The left showed the DNA molecular mass marker (lane 5); (b) RT-PCR analysis. DNA fragment was* amplified* from the transformed plant with E0 specific primers. Lane 1: PCR product from the plasmid pBI121-E0. Lane 2: RT-PCR product from nontransformed* Astragalus*. Lanes 3-4: RT-PCR product from different transgenic* Astragalus *spp. Lane 5 showed the DNA molecular mass marker.

To confirm the integration of the E0 gene into the chromosome of transgenic seedlings, genomic DNA and total RNA were isolated from transgenic* Astragalus* and tested by PCR and RT-PCR. As expected, specific bonds of 706 bp were detected in all groups except for the negative control groups (Figures 1(a) and 1(b)).

### 3.2. Expression of E0 Protein in Transgenic Astragalus Seedlings

**Figure 2:**
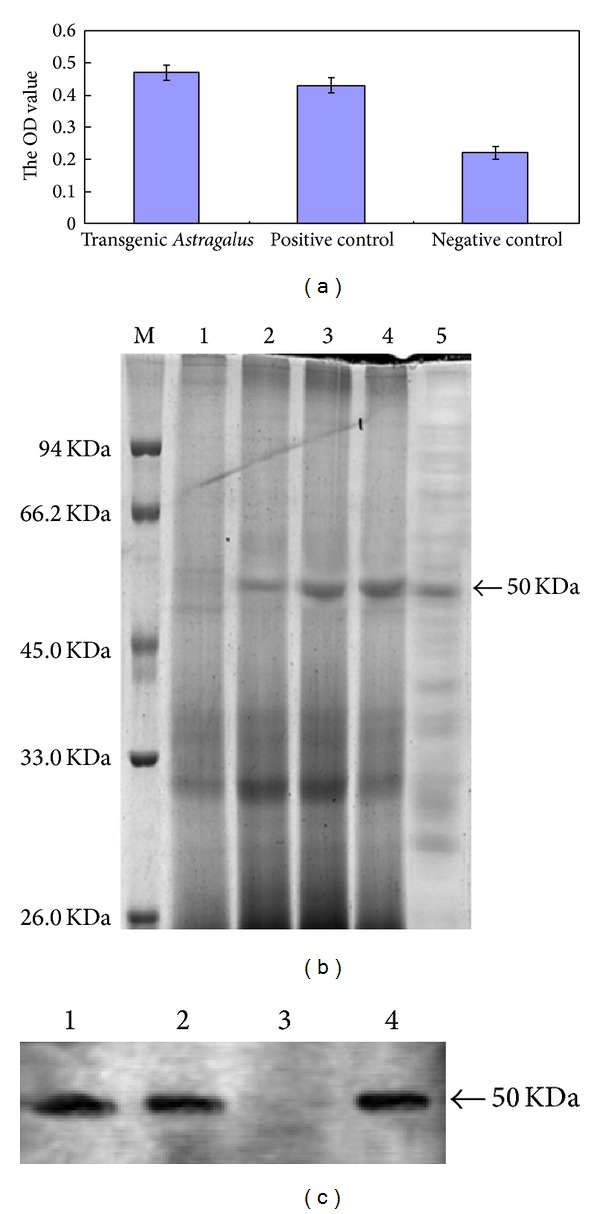
Protein analysis in transgenic* Astragalus* extract. (a) ELISA test to determine the presence of the antigen E0 in transgenic* Astragalus *leaves extract. ELISA protocol was described in methods section. Transgenic* Astragalus* group (the recombinant proteins extracted from transgenic* Astragalus* leaves); positive control (E0 protein expressed in BHK-21 cells); negative control (proteins extracted from wild-type* Astragalus*). (b) Coomassie brilliant blue staining of protein extracted from transgenic* Astragalus* leaves analyzed by 12% of SDS-PAGE. M: protein markers, 1: negative control, 2–4: transgenic* Astragalus* groups, 5: positive control. (c) Western blot analysis showing immune activity of the E0 protein expressing in transgenic* Astragalus*. In transgenic* Astragalus* group (lanes 1, 4) and positive control (lane 2 E0 protein expressed in BHK-21 cells) a specific band of 50 kDa was detected. Lane 3: negative control (proteins extracted from wild-type* Astragalus*).

The ELISA assay was used to determine the expression of glycoprotein E0 in transgenic* Astragalus*. The OD490 values of transgenic* Astragalus* groups were similar to the positive groups but significantly higher than the negative groups (Figure 2(a)). That reveals glycoprotein E0 was highly expressed in transgenic* Astragalus.* To further confirm the immune activity of E0 protein, the Western blot analysis was carried out. A specific band of 50 kDa corresponding to glycoprotein E0 was detected in coomassie blue gel analysis (Figure 2(b)) and Western blot for both the samples and the positive control was shown in Figure 2(c). As expected, it was not detected in the negative controls.

### 3.3. Detection of Deer Serum Antibody and Cellular Immune Level

**Figure 3:**
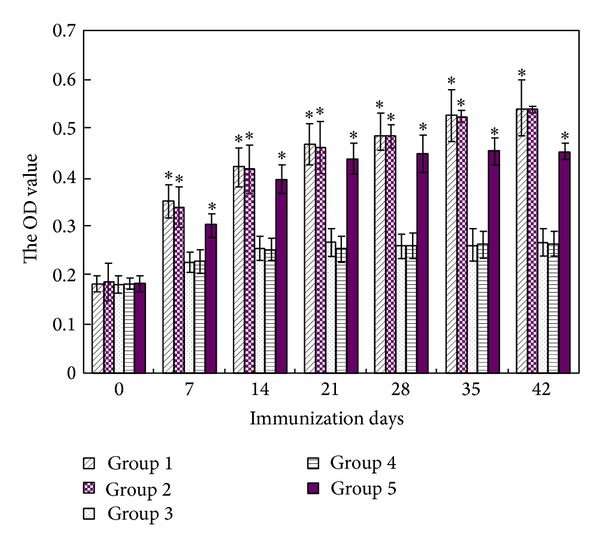
Specific humoral response in deer following vaccination. The deer were immunized subcutaneously with transgenic* Astragalus* oil vaccine (group 1), transgenic* Astragalus* aqueous vaccine (group 2), oil negative control (group 3), aqueous negative control (group 4), and inactivated BVDV vaccine (group 5). Serum samples used to assess the humoral immune response were collected on days 0, 7, 14, 21, 28, 35, and 42 after primary immunization and detected at 490 nm. *The difference between aqueous negative control (group 4) and other groups at the same days after primary immunization is significant (*P* < 0.05).

**Figure 4:**
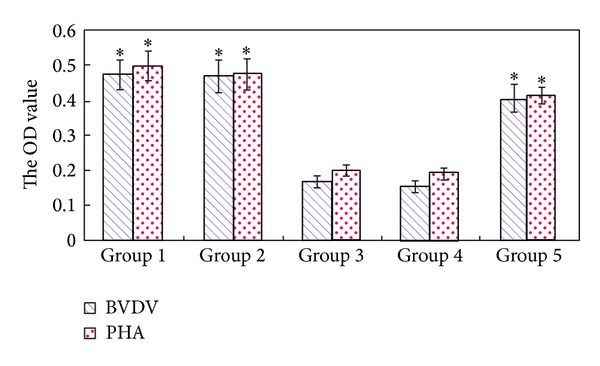
Lymphocyte blastogenesis assay. The blood was collected from each deer on day 42 after the immunization. Group 1: transgenic* Astragalus* oil vaccine; group 2: transgenic* Astragalus* aqueous vaccine; group 3: oil negative control; group 4: aqueous negative control; group 5: inactivated BVDV vaccine. *The difference between aqueous negative control (Group 4) and other groups is significant (*P* < 0.05).

The serum antibody levels of immune deer were detected by ELISA and the results are shown in Figure 3. Before immunization, no significant difference was found among all groups. But, in the serum of immunized deer for the first seven days, the serum antibodies in oil vaccine group, aqueous vaccine group, and inactivated BVDV vaccine group were significantly higher than negative groups (*P* < 0.05). The antibody levels increased with the immune time in all groups, except for negative groups. With the increasing of the immune time, the antibody levels of deer BVDV inactivated vaccine group reached the peak value after 35 days of immunization, while oil vaccine group and aqueous vaccine group reached the peak value after 42 days. This revealed that oil vaccine and aqueous vaccine gave the deer longer protection from BVDV infection.

To evaluate the cell-mediated immune responses, blood samples were collected on the 42nd day after immunization and tested for lymphocyte proliferative responses. As shown in Figure 3, the results indicated that lymphocytes present in blood from deer that received transgenic* Astragalus* plants proliferated substantially upon restimulation with BVDV antigens (Figure 3). This specific proliferation was absent in the negative groups that received nontransformed leaves (Figure 3). The result is corresponding to the result of PHA-induced lymphocyte proliferation. This suggested that BVDV-E0 specific prolonged cell-mediated immune responses in deer subcutaneously immunized with E0 transgenic* Astragalus* plant leaves were present.

## 4. Discussion

BVDV infection is an important cause of morbidity and economical losses in cattle worldwide. It is estimated that BVD generates a negative economic impact in dairy operations (between $20 and $160 per adult cow per year) [36]. Developing effective and inexpensive vaccines is critical for protecting animals against BVDV infection. With the development of genetic molecular biology and plant biotechnology, plants have emerged as a new platform for the production and delivery of antigenic proteins as plant-based vaccines. Plant-based vaccines offer several advantages over traditional vaccine such as ease of delivery, mucosal efficacy, safety, rapid scalability, and low cost. However, there are still no reports about the expression of E0 glycoprotein in plants. This experiment successfully acquired glycoprotein E0 of BVDV in transgenic* Astragalus *through pollen-tube pathway. This is the first report on the expression of E0 glycoprotein in a medicinal plant.

The glycoprotein E0 has several functions, such as virus attachment and entry to target cells and the production of neutralizing antibodies, as well as having effects on the pathogenicity of BVDV [37, 38]. The glycoprotein E0 consists of 227 amino acids and isoelectric point is 7.61 [39]. As a conserved protein, E0 shows less antigenic diversity than E2. However, as antigen for BVDV, E0 still has many defects. For example, E0 has many glycosylation sites that are important for proper folding and activity maintenance of protein, and it also exhibits weak immunogenicity. In this study, the transgenic* Astragalus* was successfully used as a platform for production of the glycoprotein E0, which allowed us to overcome the above shortcomings.* Astragalus* extracted as vaccine adjuvant significantly improves immune activity of E0 subunit vaccine. As such, the plant-made glycoprotein E0 has the ability to generate an immune response in sika deer. This study provides a new method for proteins with weak immunogenicity to be used as transgenic vaccine candidates by use of a plant system.

The use of transgenic plants as new antigen-delivery systems for subunit vaccines has been increasingly explored [40].* Astragalus* has immunomodulatory effects and* Astragalus* as host plants can play a role in the immune adjuvant to enhance the immune level. Plants can be mass-produced, inexpensive source of antigens, and an ideal system for subunit vaccine. The research provided new ideas for the development of transgenic medicinal plants vaccines.

## 5. Conclusions

In summary, glycoprotein E0 was effectively expressed in transgenic* Astragalus*. Deer immunized subcutaneously (s.c.) with the transgenic plant vaccine could develop specific humoral and cell-mediated immune responses against BVDV. The research provides new ideas for the development of medicinal plants vaccine.
